# Reactive oxygen species-mediated senescence is accelerated by inhibiting Cdk2 in Idh2-deficient conditions

**DOI:** 10.18632/aging.102259

**Published:** 2019-09-10

**Authors:** Unbin Chae, Jeen-Woo Park, Sang-Rae Lee, Hong Jun Lee, Hyun-Shik Lee, Dong-Seok Lee

**Affiliations:** 1 School of Life Sciences, BK21 Plus KNU Creative BioResearch Group, Kyungpook National University, Daegu, Republic of Korea; 2 School of Life Sciences and Biotechnology, College of Natural Sciences, Kyungpook National University, Daegu, Republic of Korea; 3 Futuristic Animal Resource and Research Center, Korea Research Institute of Bioscience and Biotechnology (KRIBB), Cheongju-si, Chungcheongbuk-do, Republic of Korea; 4 National Primate Research Center, Korea Research Institute of Bioscience and Biotechnology (KRIBB), Cheongju-si, Chungcheongbuk-do, Republic of Korea; 5 College of Medicine, Chungbuk National University, Cheongju-si, Chungcheongbuk-do, Republic of Korea; 6 Department of Radiology, Chungbuk National University Hospital, Cheongju-si, Chungcheongbuk-do, Republic of Korea; 7 Research Institute, e-biogen Inc., Seoul, Republic of Korea

**Keywords:** isocitrate dehydrogenase 2 (IDH2), reactive oxygen species (ROS), cyclin-dependent kinase 2 (Cdk2), senescence, cell cycle

## Abstract

Among the many factors that promote cellular senescence, reactive oxygen species (ROS) are a focus of intense research because of their critical role in accelerating cellular senescence and initiating senescence-related diseases that can be fatal. Therefore, maintaining the proper balance of ROS in cells is a key method to alleviate senescence. Recent studies have found that isocitrate dehydrogenase 2 (IDH2), a critical enzyme of the tricarboxylic acid cycle, participates in ROS generation and in cellular dysfunction that is induced by excessive levels of ROS. Loss of IDH2 induces mitochondrial dysfunction that promotes excessive ROS generation and the development of several diseases. The results of this study suggest that Idh2 plays an important role in cellular senescence. Idh2 deficiency resulted in senescence-associated phenotypes and increased levels of senescence marker proteins in mouse embryonic fibroblasts and tissues. Furthermore, excessive ROS were generated in Idh2-deficient conditions, promoting cellular senescence by inducing cell cycle arrest through cyclin-dependent kinase 2. These results indicate that loss of Idh2 is a critical factor in regulating cellular senescence. Taken together, our findings contribute to the field of senescence research and suggest that IDH2 is a potential target of future anti-senescence studies.

## INTRODUCTION

Senescence, also called biological aging, is a functional degradation process of cells and tissues [1], and oxidative stress and free radicals are two critical elements that induce senescence [2]. Accumulation of oxidative stress in cells and tissues causes several age-related diseases that shorten the life span of the organism [3]. Reactive oxygen species (ROS) are a byproduct of normal mitochondrial respiration, specifically the oxidative phosphorylation pathway that activates the production of various superoxides [4]. Superoxide dismutase converts superoxides to hydrogen peroxide (H_2_O_2_), which participates in cellular signaling pathways. Maintaining the balance of ROS formation is important for cellular homeostasis [5]. Excessive ROS formation can induce oxidative stress, which plays a critical role in the development of various diseases, including aging [6].

Isocitrate dehydrogenase (IDH) is a highly conserved enzyme that catalyzes isocitrate to α-ketoglutarate (α-KG) [7]. IDH is classified into three isotypes, partly based on cellular localization. IDH1 is found in the cytosol and IDH2 and IDH3 are found in mitochondria. Many studies have focused on IDH2 because of how it functions in mitochondria. IDH2 plays an important role in maintaining mitochondrial redox balance by supplying NAPDH for NADPH-dependent antioxidant enzymes [8]. A recent study suggested that the presence or absence of IDH2 has a critical role in various diseases [9]. Many studies point to IDH2 dysfunction-induced ROS as the main component of disease states. IDH2 deficiency induces a mitochondrial redox imbalance that generates excessive ROS and oxidative stress. As a result, IDH2 deficiency-induced ROS imbalances promote diseases including liver failure [9], cardiac hypertrophy [10], Parkinson’s disease [11], metabolic disorders [12], renal dysfunction [13], hearing loss [14] and kyphosis [15].

Many studies already suggest that ROS are critical elements of accelerated aging [16]. Therefore, regulating excessive ROS and oxidative stress is a compelling target for anti-aging therapies. Previous studies suggested that downregulation of IDH2 is one of the critical factors of ROS generation [17], suggesting that IDH2 could be a potential target for slowing the aging process. In this study, we used mouse embryonic fibroblasts (MEFs) to study senescence. We determined that Idh2 expression is downregulated in senescence-induced MEFs. Furthermore, Idh2 deficiency accelerated senescence-associated phenotypes in MEFs. In conclusion, we suggest that IDH2 gene expression is a crucial regulator of aging and can be a potential target for future anti- aging experiments.

## RESULTS

### Idh2 expression is decreased in senescent MEFs and tissues

**Figure 1:**
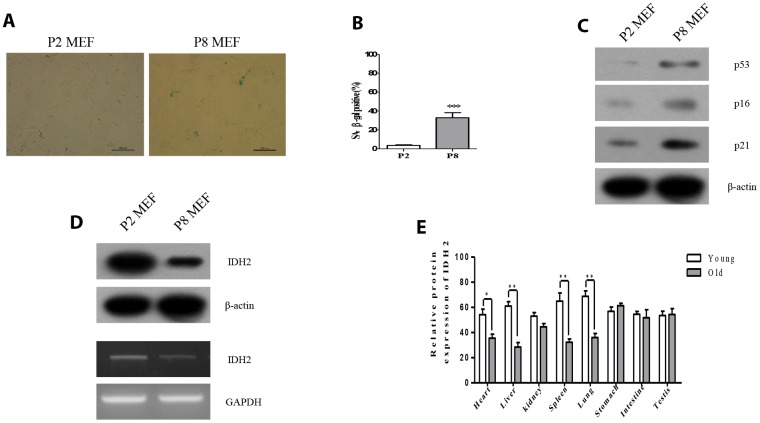
**Idh2 was downregulated in senescent mouse embryonic fibroblasts (MEFs) and aging tissues.** (**A**) SA-ß-gal staining of Passage 2 (P2) and Passage 8 (P8) MEFs. Scale bar, 200 µm. (**B**) Statistical analysis of SA-ß-gal-stained positive cells between P2 and P8 MEFs. (**C**) Western blot analysis between P2 and P8 MEFs. The following antibodies were used: anti-p53, anti-p16, anti-p21, and anti-ß-actin. (**D**) Detection of Idh2 expression level in P2 and P8 MEFs. Western blotting and reverse-transcriptase PCR was performed for detecting Idh2. (**E**)Relative protein expression of Idh2 was detected in mouse tissues using western blot analysis. Ten-week-old and 47-week-old mice were used. Data are expressed as means ± SD (*n* = 3). *p < 0.05, **p < 0.01, and ***p < 0.001.

To measure the expression levels of Idh2 in senescence-induced MEFs and aged mouse tissues, we first evaluated the senescence-associated β-gal activity (SA-β-gal), an established hallmark of cellular senescence. Passage 8 (P8) MEFs exhibit higher SA-β-gal staining than Passage 2 (P2) MEFs (Figure 1A and 1B). Levels of well-known senescence marker proteins (p53, p16, and p21) are also increased in P8 MEFs (Figure 1C). Next, we confirmed the expression level of Idh2 in P8 MEFs. Protein and mRNA levels of Idh2 are decreased in P8 MEFs (Figure 1D). *Idh2* mRNA expression was also measured using glucose-6-phosphate dehydrogenase (*G6PD*) as a control to confirm downregulation of *Idh2* in P8 MEFs (Supplementary Figure 1). Idh2 levels are also decreased significantly in heart, liver, spleen, and lung tissues from old mice (Figure 1E). From these results, we conclude that expression levels of Idh2 are decreased in p8 MEFs and aged mouse tissues.

### Downregulation of Idh2 accelerates senescence in MEFs

Because Idh2 expression is decreased in P8 MEFs, we hypothesized that inhibiting Idh2 expression affects MEF senescence. We decreased *Idh2* expression by transfecting small interfering RNA (siRNA) into MEFs and by *Idh2* knockout in mice.

**Figure 2:**
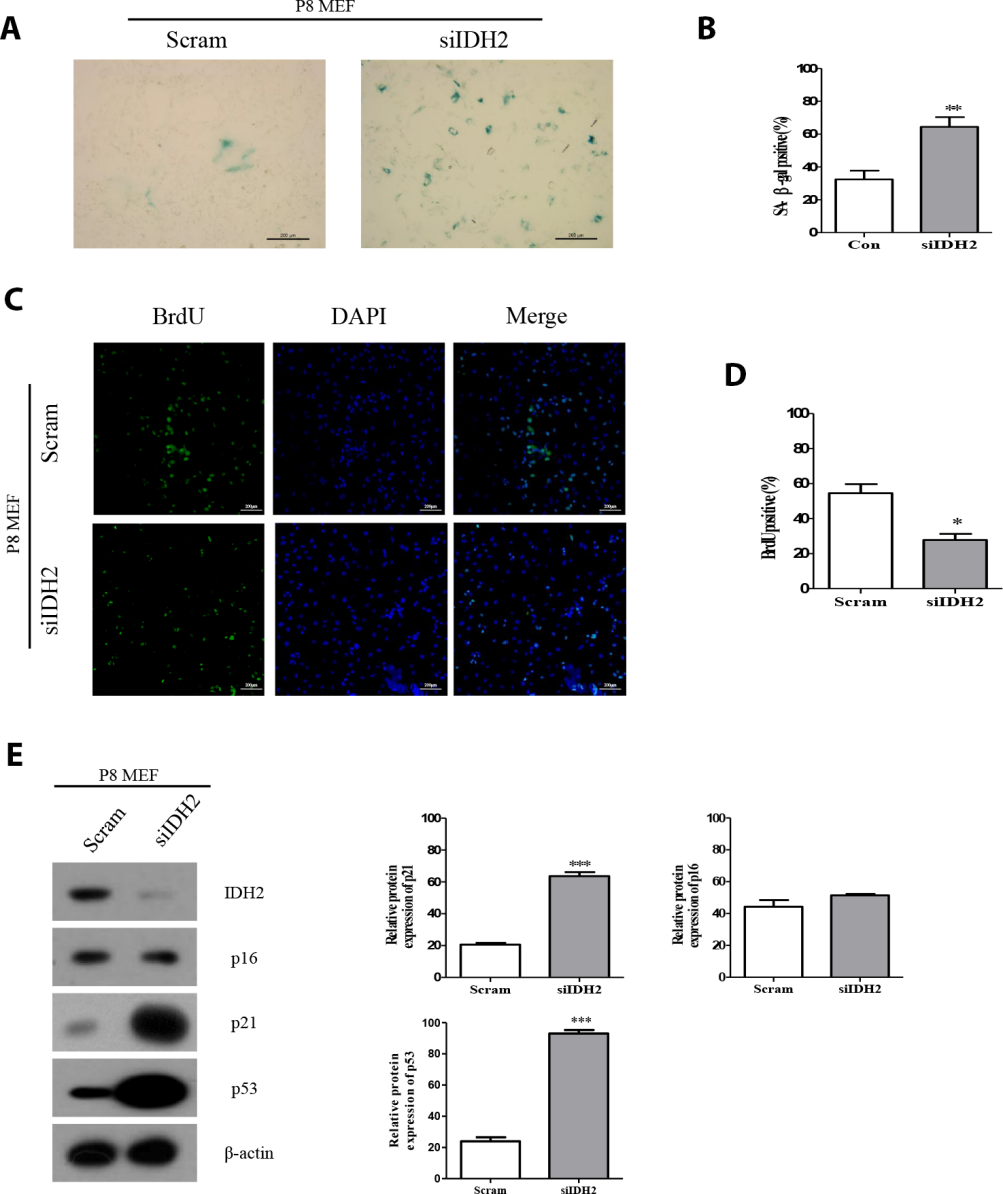
**Downregulation of Idh2 in senescent mouse embryonic fibroblasts (MEFs) accelerates the senescence phenotype.** (**A**) SA-β-gal staining of control and Idh2-silenced MEFs. Passage 8 (P8) MEFs were used. Scale bar, 200 μm. (**B**) Statistical analysis of SA-β-gal-stained positive cells between control and *Idh2*-knockdown MEFs. (**C**) BrdU levels between control and *Idh2*-knockdown MEFs as determined by immunocytochemistry. Nuclei were stained with DAPI, and the merged images show BrdU and DAPI signals. Scale bar, 10 μm. (**D**) Statistical analysis of BrdU-positive cells between control and *Idh2*-knockdown MEFs. (**E**) Western blot analysis between control and *Idh2*-knockdown MEFs. The following antibodies were used for detection: anti-Idh2, anti-p16, anti-p21, anti-p53, and anti- β-actin. Data are expressed as means ± SD (*n* = 3). *p < 0.05, **p < 0.01, and ***p < 0.001.

Downregulation of *Idh2* expression using siRNA (also referred to as “*Idh2* knockdown”) results in a marked increase of SA-β-gal staining compared with the level of SA-β-gal staining in response to transfection with scrambled siRNA (Figure 2A and 2B, respectively). Immunofluorescence staining using 5-bromo-2′-deoxyuridine (BrdU) in MEFs revealed that cell proliferation is significantly inhibited in *Idh2*-knockdown MEFs (Figure 2C and 2D). Next, we measured the level of senescence-related marker proteins in control and *Idh2*-knockdown MEFs. Interestingly, *Idh2*-knockdown MEFs show increased levels of p21 and p53, but not p16 (Figure 2E).

**Figure 3:**
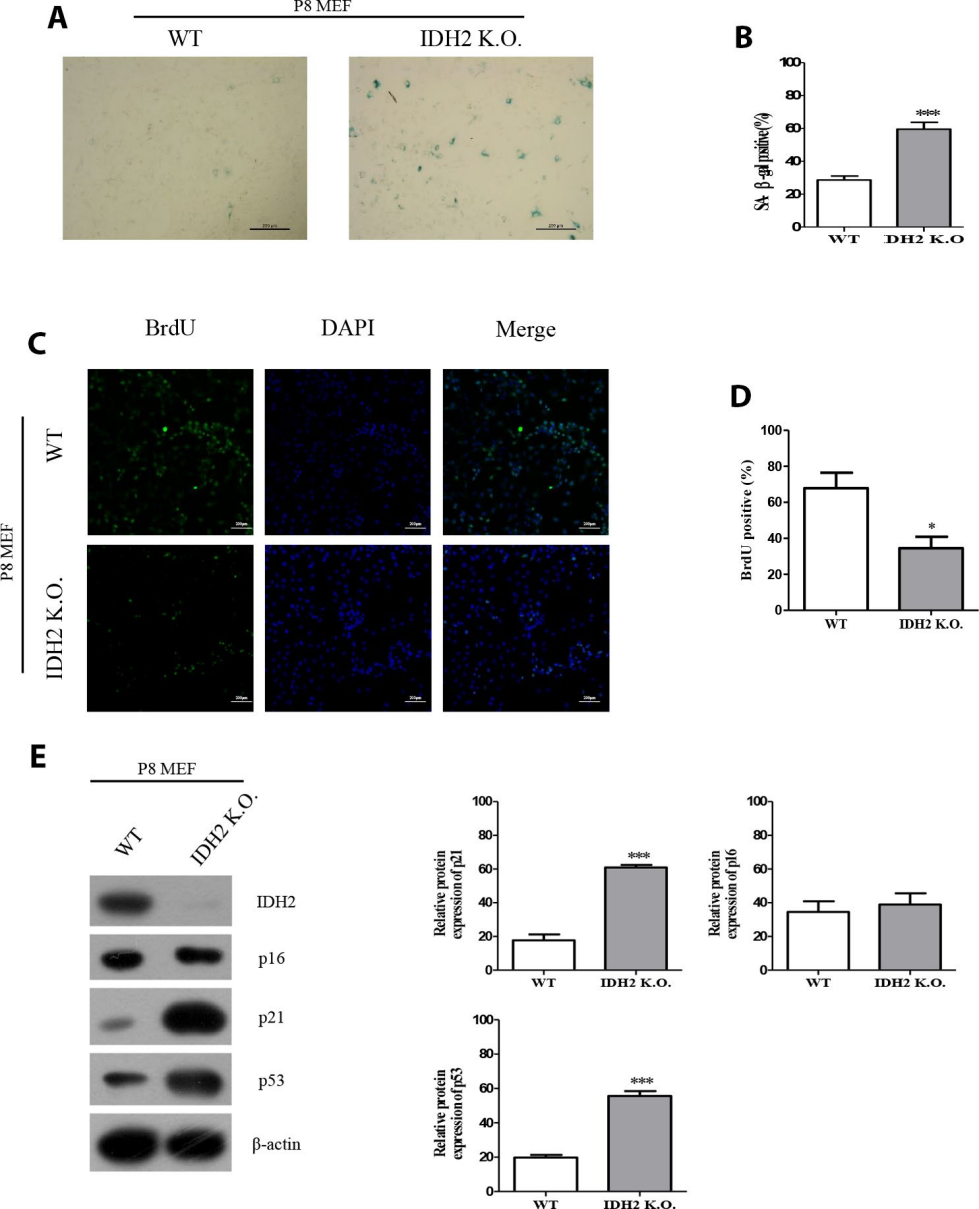
**The senescence phenotype is promoted in *Idh2* knockout mouse embryonic fibroblasts (MEFs).** (**A**) SA-β-gal staining of wild type and *Idh2* knockout MEFs. Passage 8 (P8) MEFs were used. Scale bar, 200 μm. (**B**) Statistical analysis of SA-β-gal-stained positive cells between wild type and *Idh2* knockout MEFs. (**C**) BrdU level between wild type and *Idh2* knockout MEFs as determined by immunocytochemistry. Nuclei were stained with DAPI, and the merged images show BrdU and DAPI signals. Scale bar, 10 μm. (**D**) Statistical analysis of BrdU-positive cells between wild type and *Idh2* knockout MEFs. (**E**) Western blot analysis between wild type and *Idh2* knockout MEFs. The following antibodies were used for detection: anti-Idh2, anti-p16, anti-p21, anti-p53, and anti-β-actin. Data are expressed as means ± SD (*n* = 3). *p < 0.05, **p < 0.01, and ***p < 0.001.

Next, we used *Idh2* knockout mice to evaluate senescence in mice that do not express *Idh2*. MEFs from *Idh2* knockout mice (Idh2 knockout MEFs) show a significant increase in SA-β-gal staining compared to that in MEFs from wild type mice (wild type MEFs) (Figure 3A and 3B). BrdU labeling is also decreased in *Idh2* knockout MEFs compared to that in wild type MEFs (Figure 3C and 3D). In *Idh2* knockout MEFs, levels of senescence marker proteins p21 and p53 are increased, but p16 levels are not (Figure 3E), which is the same result seen with *Idh2*-knockdown MEFs. In conclusion, downregulation of *Idh2* accelerates senescence through p53 and p21 signaling.

### *Idh2* knockout mice show unusual histopathology in lung and spleen tissue

**Figure 4:**
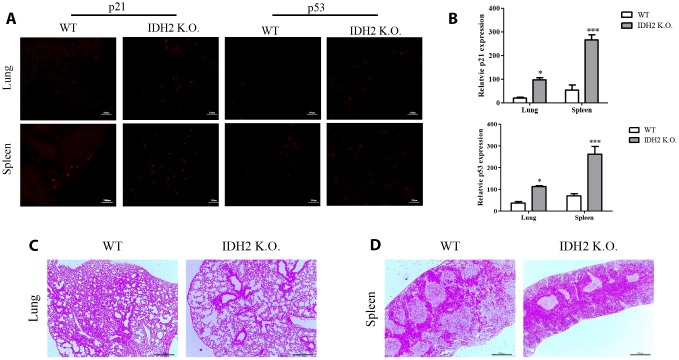
**Acceleration of senescence in several tissues is observed in *Idh2* knockout mice.** (**A**) P21 and p53 levels in lung and spleen tissues from wild type and *Idh2* knockout mice as determined by immunohistochemistry. Images show p21 and p53 signals. Scale bar, 10 μm (n = 6/group). (**B**) Statistical analysis of p21 and p53-positive signals between lung and spleen tissues from wild type and *Idh2* knockout mice. (**C**) Representative H&E-stained sections from wild type and *Idh2* knockout mouse lungs (n = 6/group). (**D**) Representative H&E-stained sections from wild type and *Idh2* knockout mouse spleens (n = 6/group).

In addition to accelerating senescence via p53 and p21 signaling, *Idh2* knockout causes histopathology differences in mice. We first examined p21 levels in *Idh2* knockout and wild type mice by immunohistochemistry. Among the tissues examined, lung and spleen exhibit significantly upregulated p21 and p53 expression in *Idh2* knockout mice (Figure 4A and 4B). Hematoxylin and eosin staining (H&E staining) was used to detect histopathological changes. Lungs of *Idh2* knockout mice exhibit significantly more alveolar wall destruction than lungs from wild type mice (Figure 4C). Spleens of *Idh2* knockout mice also exhibit a reduction in white pulp, which consists of lymphoid tissue (Figure 4D). Therefore, *Idh2* knockout accelerates senescence in lung and spleen tissues through upregulation of p21 expression.

### Idh2 deficiency induces ROS generation and increases p21 expression

**Figure 5:**
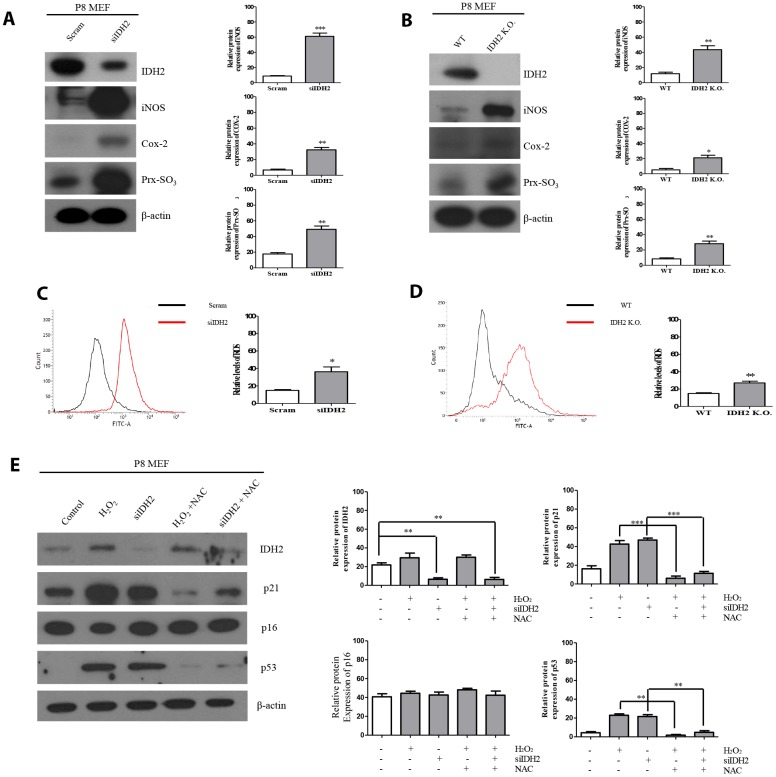
***Idh2* deficiency-mediated reactive oxygen species (ROS) generation activates p21 expression.** (**A**) Western blot analysis of pro-inflammatory mediators and oxidative stress marker proteins in control and *Idh2*-knockdown mouse embryonic fibroblasts (MEFs). The following antibodies were used for detection: anti-Idh2, anti-iNOS, anti-Cox-2, anti-Prx-SO_3_, and anti-β-actin. (**B**) Western blot analysis of pro-inflammatory mediators and oxidative stress marker proteins between wild type and *Idh2* knockout MEFs. The following antibodies were used for detection: anti-Idh2, anti-iNOS, anti-Cox-2, anti-Prx-SO_3_, and anti-β-actin. (**C**) Control and *Idh2*-knockdown MEFs were incubated with DCF-DA for 15 min at 37**°**C and intracellular ROS levels were analyzed by flow cytometry. (**D**) Wild type and *Idh2* knockout MEFs were incubated with DCF-DA for 15 min at 37**°**C and intracellular ROS levels were analyzed by flow cytometry. (**E**) After transfecting MEFs with siIdh2, H_2_O_2_ was added for 3 days. NAC was added 4 h before H_2_O_2_ treatment. Western blot analysis was detected with the following antibodies. Data are expressed as means ± SD (*n* = 3). *p < 0.05, **p < 0.01, and ***p < 0.001.

Previous studies demonstrated that excessive ROS production accelerates the aging process and IDH2 deficiency elevates ROS generation. However, there have been no studies on the relationship between IDH2 deficiency-induced ROS generation and aging. Our studies suggest that *Idh2* deficiency accelerates aging through the p21 signaling pathway. Therefore, we used siRNA and *Idh2* knockout MEFs to downregulate *Idh2* expression. Before these experiments, we evaluated off-target effects for the Idh2 siRNA sequence by testing another predesigned siRNA (Supplementary Figure 2). All siRNAs show similar expression patterns of p21, p53, and p16 in MEFs. *Idh2* knockdown in MEFs results in increased levels of pro-inflammatory mediators, iNOS and Cox-2, and a well-known ROS marker protein, Prx-SO_3_ (Figure 5A). *Idh2* knockout MEFs also show increased levels of iNOS and Cox-2 and Prx-SO_3_ (Figure 5B). Furthermore, increased levels of total ROS are seen in *Idh2*-knockdown and *Idh2* knockout MEFs using flow cytometry (Figure 5C and 5D, respectively). Next, we used H_2_O_2_ to detect changes in ROS levels in P8 MEFs (Figure 5E). H_2_O_2_ is a well-known ROS-generating material that induces premature senescence. H_2_O_2_ treatment increases levels of senescence marker proteins p21 and p53. Furthermore, an ROS scavenger, N-acetyl cysteine (NAC), prevents the upregulation of senescence and ROS marker proteins. *Idh2* knockdown also results in a similar pattern as H_2_O_2_ treatment in MEFs. NAC treatment also prevents *Idh2* knockdown-induced upregulation of senescence marker proteins. We conclude that Idh2 downregulation induced ROS generation, which promoted an increase in p21 expression.

### The absence of *Idh2* promotes senescence by inhibiting cyclin-dependent kinase 2

**Figure 6:**
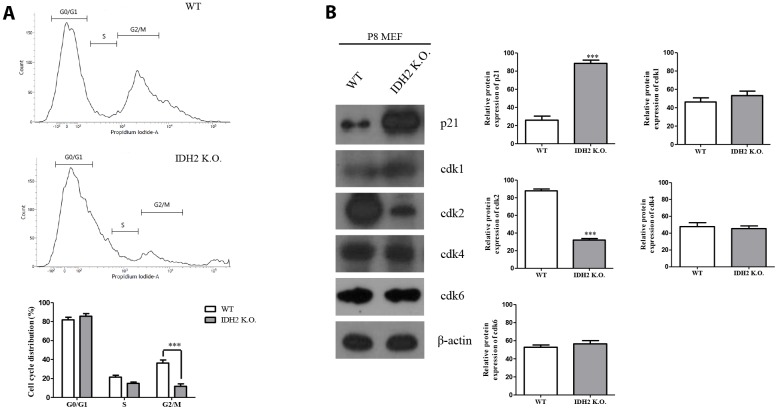
**Downregulation of *Idh2* promotes senescence in mouse embryonic fibroblasts (MEFs) through suppressing Cdk2.** (**A**) Wild type and *Idh2* knockout MEFs were incubated with propidium iodide for 15 min at 37**°**C. Cell cycle was detected with flow cytometry. (**B**) Cyclin-dependent kinase protein levels were detected with western blot analysis in wild type and *Idh2* knockout MEFs. The following antibodies were used for detection: anti-p21, anti-Cdk1, anti-Cdk2, anti-Cdk4, anti-Cdk6, and anti-β-actin. Data are expressed as means ± SD (*n* = 3). *p < 0.05, **p < 0.01, and ***p < 0.001.

Generally, the induction of senescence is associated with cell cycle regulation. Cyclin-dependent kinase proteins participate in regulating the cell cycle, including Cdk1, Cdk2, Cdk4, and Cdk6. Multiple studies have suggested that dysfunction of cyclin-dependent kinase proteins induces cell cycle arrest, which can promote cellular senescence [18]. Therefore, we examined cell cycle arrest and dysfunction of cyclin-dependent kinase proteins in *Idh2* knockout mice by propidium iodide staining to evaluate cell cycle by flow cytometry. Compared with wild type MEFs, *Idh2*-knockout MEFs show a low percentage of cell cycle distribution in G0 and G1 phases (Figure 6A). Furthermore, cyclin-dependent kinase protein expression levels show that only Cdk2 is decreased in *Idh2* knockout MEFs (Figure 6B). In addition, we measured the *Cdk2* mRNA level in *Idh2* knockout P8 MEFs (Supplementary Figure 3). In contrast with the protein level, *Cdk2* mRNA levels are unaffected by *Idh2* knockout. Therefore, *Idh2* knockout induces cell cycle arrest through inhibiting Cdk2 at the protein level.

### Upregulation of Idh2 protects against senescence

**Figure 7:**
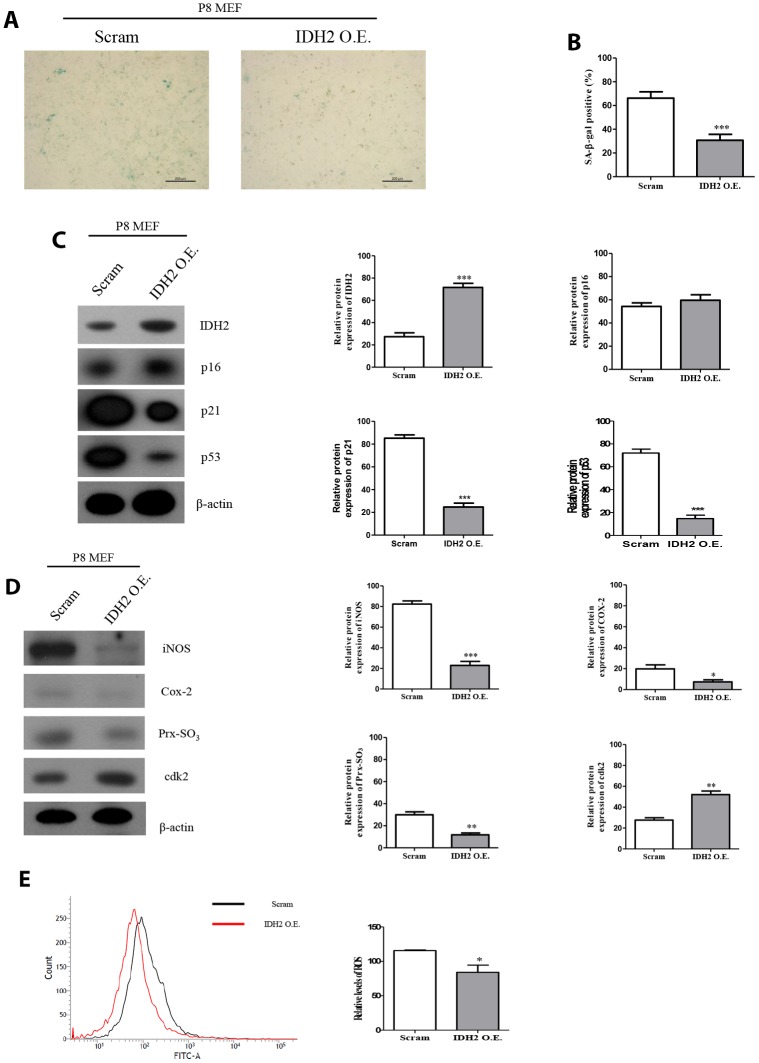
**Overexpression of *Idh2* prevents the acceleration of senescence in mouse embryonic fibroblasts (MEFs).** (**A**) SA-β-gal staining of control and *Idh2*-overexpressing MEFs. pLenti 6.3 *Idh2* plasmid was transfected into Passage 8 (P8) MEFs. (**B**) Statistical analysis of SA-β-gal-stained positive cells between control and *Idh2*-overexpressing MEFs. (**C**) Senescence-associated marker proteins were detected using western blot analysis in control and *Idh2*-overexpressing MEFs. The following antibodies were used for detection: anti-Idh2, anti-p16, anti-p21, anti-p53, and anti-β-actin. (**D**) Pro-inflammatory mediators, reactive oxygen species (ROS) marker proteins, and cyclin-dependent kinase 2 were detected using western blot analysis. The following antibodies were used for detection: anti-iNOS, anti-Cox-2, anti-Prx-SO_3_, and anti-β-actin. (**E**) Relative intracellular ROS levels were detected in control and *Idh2*-overexpressing MEFs. Intracellular ROS were detected by flow cytometry. Data are expressed as means ± SD (*n* = 3). *p < 0.05, **p < 0.01, and ***p < 0.001.

Our results suggested that downregulation of *Idh2* accelerates the development of senescence through the p21 signaling pathway. We next examined whether overexpression of *Idh2* could protect against senescence. *Idh2*-overexpressing P8 MEFs show fewer SA-β-gal-positive cells than normal P8 MEFs (Figure 7A and 7B). Furthermore, the increased p21 and p53 levels seen in P8 MEFs are downregulated by overexpressing *Idh2* (Figure 7C). Senescence-related ROS generation was also inhibited in *Idh2*-overexpressing P8 MEFs. The increased levels of iNOS, COX-2, and Prx-SO_3_ seen in P8 MEFs were diminished by *Idh2* overexpression. Furthermore, *Cdk2* levels were higher in P8 MEFs that overexpress *Idh2* than in control P8 MEFs (Figure 7D). Cellular ROS levels were also decreased in *Idh2*-overexpressing P8 MEFs (Figure 7E). We conclude that upregulation of *Idh2* expression can decelerate cellular senescence.

## DISCUSSION

In this study, we examined the effects of *Idh2* deficiency in cellular senescence, which accelerates aging. *Idh2* levels are decreased in heart, liver, spleen, and lung tissues in aged mice. Using wild type and *Idh2* knockout MEFs, SA-β-gal staining and BrdU assay data suggested that *Idh2* deficiency promotes cellular senescence. Passage 8 *Idh2* knockout MEFs exhibited senescence-associated phenotypes. We confirmed that *Idh2* deficiency increased ROS generation, one of the major factors that promote cellular senescence. Furthermore, we demonstrated that *Idh2* deficiency-induced ROS promote senescence signaling through p53 and p21, thereby inhibiting Cdk2, a well-known cyclin-dependent kinase that functions in the cell cycle.

Previous studies demonstrated that ROS act on various cellular organelles and signaling pathways [19]. Excessive cellular ROS production affects mitochondria by damaging mitochondrial DNA [20]. As seen in Figure 1, we confirmed that Idh2 expression was diminished in senescence-induced conditions. Because IDH2 functions in metabolism, mitochondrial function, and ROS generation, we expected that IDH2 would be a senescence-inducing factor. ROS are related to IDH2 and are known to play a major role in accelerating senescence and accompany changes in cellular function and senescence-related diseases.

Multiple studies have already suggested that excessive ROS generation affects Ras, p53, p21, and p16 signaling pathways, which are closely related to cell senescence [21]. P53 activation triggered by excessive ROS can inhibit autophagic functions, resulting in mitochondrial dysfunction, which in turn, promotes cell senescence [22]. Therefore, maintenance of a proper balance of ROS is important for cellular homeostasis. In Figures 2 and 3, our data also supported that activation of p53 by downregulation of *Idh2* expression upregulates p21, but not p16, which promotes cellular senescence. Increased p21 expression was also detected in tissues from *Idh2* knockout mice, such as lung and spleen (Figure 4). H&E staining data also supported that *Idh2* knockout mice show age-related features such as alveolar wall destruction in lung, necrotic debris accumulation in liver and a diminished number of inflammatory cells in spleen. Furthermore, we confirmed that excessive ROS generation triggered by *Idh2* knockdown induced p53 activation, which in turn triggered the upregulation of p21 (Figure 5). Finally, upregulation of p21 expression inhibited Cdk2, which has a critical role in cell cycle regulation (Figure 6).

Additionally, we confirmed the apoptotic signaling pathway in *Idh2*-deficient conditions. Because IDH2 is one of the most critical enzymes in the tricarboxylic acid (TCA) cycle, we predicted that generating *Idh2*-deficient conditions by siRNA or *Idh2* knockout would affect apoptosis. We confirmed that neither caspase 3 nor PARP, which are well known apoptotic marker proteins in mitochondria-mediated apoptosis, were induced by *Idh2* knockdown (Supplementary Figure 4). Furthermore, non-mitochondrial apoptosis was also confirmed through Annexin V in flow cytometry. Contrary to our prediction, no significant changes in marker proteins and Annexin V were detected in *Idh2* knockdown MEFs.

In the present study, *Idh2* deficiency results in excessive ROS generation. Recent studies suggest that IDH2, a critical enzyme in the TCA cycle in mitochondria, is one of the major factors correlated with ROS-induced cellular dysfunction. Many studies support that IDH2 deficiency promotes several diseases, including cancer [23], ischemia-reperfusion injury [9], inflammation [24], and age-related spinal deformities [17]. However, the effect of IDH2 deficiency on cellular senescence and senescence-related diseases has yet to be elucidated. Through this study, we demonstrate that *Idh2* deficiency-induced ROS generation activates p53 and p21 expression, thereby inhibiting Cdk2. However, more studies are needed to examine the relationship between Idh2 deficiency and age-related diseases further.

In Figure 7, we examined whether overexpression of *Idh2* could slow the development of cellular senescence. Upregulation of *Idh2* inhibited the senescence-associated phenotype in MEFs. Furthermore, *Idh2* overexpression decreased levels of senescence-associated marker proteins, pro-inflammatory mediators, and ROS markers. Furthermore, Cdk2 expression was also elevated upon *Idh2* overexpression. However, we cannot conclusively say that upregulation of *Idh2* has an anti-senescence effect; we can only suggest the possibility that Idh2 is potentially a useful protein for studying senescence. Therefore, more studies are needed on the relationship between Idh2 and cellular senescence.

**Figure 8:**
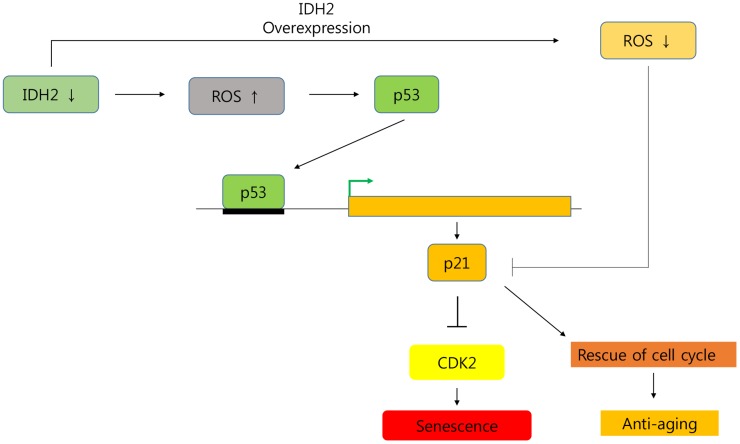
**Graphical abstract of the senescence pathway in *Idh2*-deficient conditions.** *Idh2* deficiency-induced ROS generation accelerates p53 and p21 signaling pathways which inhibit Cdk2 expression. Furthermore, overexpression of *Idh2* prevented senescence-associated phenotypes by decreasing the levels of senescence signaling pathway-associated proteins p21 and p53.

In conclusion, we demonstrated that *Idh2* expression decreased cellular senescence, and downregulation of *Idh2* promoted it. Furthermore, *Idh2* deficiency-induced cell cycle arrest by inhibiting Cdk2 through the p53 and p21 signaling pathways (Figure 8). Interestingly, overexpression of *Idh2* alleviated senescence-associated phenotypes as shown by decreased SA-β-gal staining and expression of p21 and p53. Furthermore, *Idh2* overexpression increased levels of pro-inflammatory mediators and ROS. Further studies are needed to investigate which factors decrease *Idh2* levels in age-dependent conditions. In addition, the anti-senescence effect of Idh2 should also be studied further. Although anti-aging studies have long been a popular focus for many studies, as life expectancy increases, aging studies are receiving more attention. As mentioned above, although more studies are needed, IDH2 represents a fascinating target of future cellular senescence studies.

## MATERIALS AND METHODS

### Cell culture and treatment

MEFs and NIH 3T3 cells were cultured in Dulbecco’s modified Eagle medium (Welgene, Daegu, Korea) containing 10% fetal bovine serum (Thermo Scientific, Waltham, MA, USA) and 1% penicillin/streptomycin (Welgene) and incubated at 37°C in a 5% CO_2_ incubator (SANYO, Osaka, Japan). MEFs and NIH 3T3 cells were grown to 60% confluency and then transfected with 10 pmol of siRNA against Idh2 (siIdh2; Bioneer, Daejeon, Korea) or scrambled siRNA control.

### Preparation of MEFs

All animal experiments were approved and conducted per the guidelines of the Animal Care Committee of Kyungpook National University. MEFs were generated from 10 to 12.5-day-old embryos derived from WT and Idh2 knockout mice. Embryos were minced, dispersed in 0.05% trypsin/EDTA, and incubated in 5% CO_2_ at 37°C for 30 min. After incubation, the mass was pipetted for dispersion and then allowed to settle for 1 min, after which the pellet was removed. The cell suspension was plated and incubated at 37°C until confluence. Then, confluent MEFs were stocked using stock solution (Sigma-Aldrich, St. Louis, MO, USA).

### SA-β-gal staining

SA-β-gal staining was carried out using a commercial kit (Cell signaling, Danvers, MA, USA). Before staining, the medium was removed from the cells, and cells were washed three times with phosphate-buffered saline (PBS). Then, cells were fixed for 15 min at room temperature. After fixation, cells were washed with PBS, β-galactosidase staining solution was added, and cells were incubated at 37°C overnight in a dry incubator without CO_2_. Stained cells were observed with a light microscope.

### BrdU assay

BrdU (Thermo Scientific) was dissolved as a 10 mM solution in DMSO (Sigma). BrdU solution was then diluted to 10 μM in cell culture medium. Culture medium was removed and replaced with BrdU labeling solution. Cells were incubated at 37°C for 2 hours. After incubation, cells were fixed using 3.7% paraformaldehyde. BrdU was labeled by an anti-BrdU primary antibody (SantaCruz, Dallas, TX, USA) at room temperature overnight. After labeling with an appropriate secondary antibody, BrdU was detected using a confocal microscope (Carl Zeiss, Oberkochen, Germany).

### Western blot analysis

Whole-cell lysates were obtained using PRO-PREP protein extraction solution (Intron Biotechnology, Gyeonggi-do, Korea). Proteins were separated by SDS-PAGE, and bands were transferred to nitrocellulose membranes (Pall Corporation, Pensacola, FL, USA). The membranes were then incubated with the following primary antibodies at 4°C overnight: anti-p53, anti-Idh2, anti-B-actin, anti-Cdk1, anti-Cdk2, anti-Cdk4, anti-Cdk6, anti-iNOS, anti-Caspase3, and anti-PARP (Cell signaling); anti-p16 and anti-p21 (Abcam, Cambridge, UK); anti-COX-2 (SantaCruz); and anti-Prx-SO3 (Ab Frontier, Seoul, Korea). After washing with buffer, the membranes were incubated with the appropriate secondary antibody (Thermo Scientific) at room temperature for 6 hours.

### RNA isolation and RT-PCR

Total RNA was isolated using TRI-Reagent (Invitrogen, Carlsbad, CA, USA). cDNA was synthesized using Reverse Transcription Premix (Bioneer). PCR was performed using gene-specific primers and PCR premix (Bioneer). The following primers were used: Idh2, 5′-ATCAAGCAGAAGCTCATCCTGC-3′ (forward) and 5′-TCTGTGGCCTTGTACTGGTCG-3′ (reverse); Idh1, 5′-GTCGTCATGCTTATGGGGAT-3′ (forward) and 5′-CAACACCACCACCTTCTTCA-3′ (reverse); Idh3, 5′-ACCTTATAGCAAACACGGCG-3′ (forward) and 3′-TCTTATCATTCCCCACATTCCC-3′ (reverse); Cdk2, 5′-GCTTTCTGCCATTCTCATCG-3′ (forward) and 5′-GTCCCCAGAGTCCGAAAGAT-3′ (reverse); G6PD, 5′-TGAGGGTCGTGGGGGCTATTTTGA-3′ (forward) and 5′-GCATCAGGGAGCTTCACATTCTTG-3′ (reverse); GAPDH, 5′-ACCACAGTCCATGCCATCAC-3′ (forward) and 5′-TCCACCACCCTGTTGCTGTA-3′ (reverse). Multi-Gauge software (Fujifilm, Japan) was used to analyze band intensity.

### Immunohistochemistry

Before tissue collection from mice, animals were perfused. After tissue collection, all tissues were fixed in 10% formalin at 4°C overnight. Tissues were cryosectioned using an HM525 NX Cryostat (Thermo Scientific). Sectioned tissues were incubated with an anti-p21 and anti-p53 antibody (Abcam) at 4°C overnight. Tissues were then incubated with Alexa 549 goat anti-rabbit secondary antibody (Thermo Scientific) at 4°C overnight. Images were obtained using an LSM-710 confocal microscope (Carl Zeiss).

### Measurement of ROS and cell cycle

Total ROS levels were assessed using DCF-DA (Invitrogen, Carlsbad, CA, USA), and cell cycle determination was performed using propidium iodide (PI; BD Bioscience, Franklin Lakes, NJ, USA). Trypsinized cells were incubated with 5 μM DCF-DA and PI at 37°C for 15 min, washed with PBS, and then analyzed with a flow cytometer (BD Bioscience, San Jose, CA, USA).

### Statistical analysis

Data were shown as the mean and standard deviation (SD) of three independent experiments (n = 3). Differences observed between experimental groups were tested for statistical significance using one-way and two-way ANOVA with GraphPad Prism software. A p-value of < 0.05 was deemed statistically significant and is shown in the figures by an asterisk. P-values of < 0.01 and < 0.001 are shown by two and three asterisks, respectively.

## Supplementary Material

Supplementary Figures
