# Supplementary material for: Reactive oxygen species-mediated senescence is accelerated by inhibiting Cdk2 in Idh2-deficient conditions
Source: Aging (Albany NY). 2019 Sep 10;11(17):7242–56. doi: 10.18632/aging.102259 (PMC6756887; doi:10.18632/aging.102259)
Supplement: Supplementary Figures [file aging-11-102259-s001.pdf]

SUPPLEMENTARY FIGURES

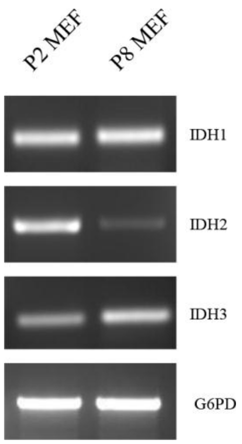

Supplementary Figure 1. G6PD used as a control for mRNA levels of *Idh1*, *Idh2*, and *Idh3*. mRNA levels of *Idh1*, *Idh2*, and *Idh3* in Passage 2 (P2) and Passage 8 (P8) mouse embryonic fibroblasts (MEFs) were detected by reverse transcriptase PCR (RT-PCR). Glucose-6-phosphate dehydrogenase (G6PD), a well-known enzyme that participates in the pentose phosphate pathway, was used as a control for analyzing relative *Idh2* mRNA levels.

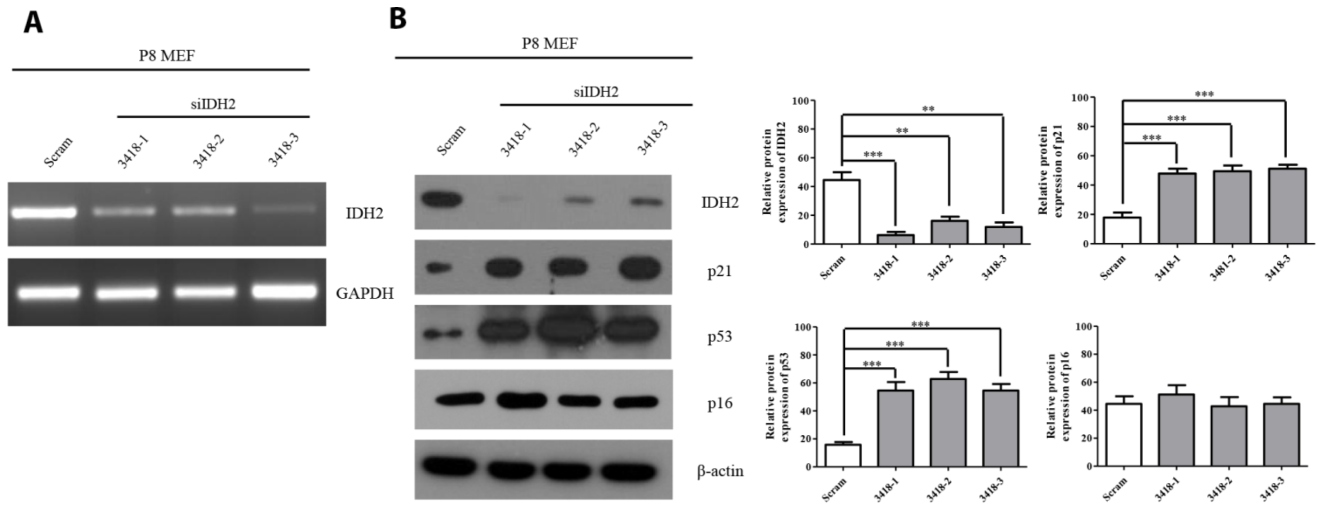

Supplementary Figure 2. Evaluating the off-target effects of Idh2 siRNA in mouse embryonic fibroblasts (MEFs). Reverse transcriptase PCR (RT-PCR) analysis (**A**) and Western blot analysis (**B**) of *Idh2* mRNA and Idh2 protein expression, respectively, in *Idh2*-knockdown MEFs. RNA and protein were extracted from Passage 8 (P8) MEFs. Commercial predesigned siRNAs (3418-1, 3418-2, 3418-3) were used for silencing of *Idh2*. RNAimax (Thermo Scientific) was used for transfection of predesigned siRNA (10 pmol). GAPDH and β-actin were used as controls for quantitative analysis of *Idh2* mRNA expression and Idh2 protein levels. The following antibodies were used for protein detection: anti-Idh2, anti-p16, anti-p21, anti-p53, and anti-β-actin. Data are expressed as means ± SD ($n = 3$). *$p < 0.05$, **$p < 0.01$, and ***$p < 0.001$.

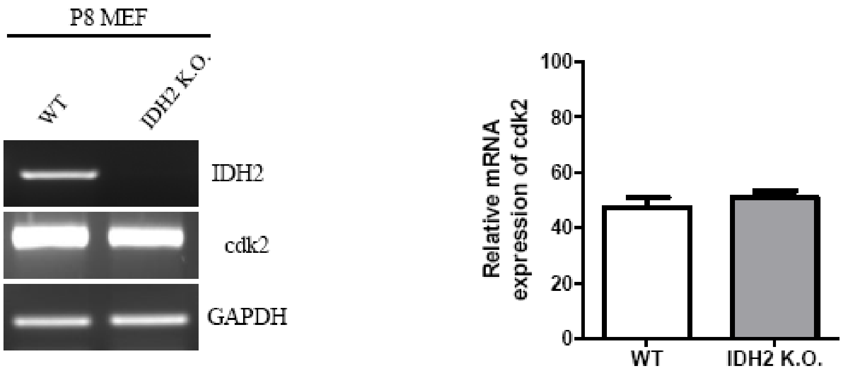

Supplementary Figure 3. Downregulation of *Idh2* did not affect the regulation of *Cdk2* mRNA. Detection of *Cdk2* mRNA expression in Passage 8 (P8) wild type and *Idh2* knockout mouse embryonic fibroblasts (MEFs). Reverse transcriptase PCR (RT-PCR) was used to detect *Cdk2* expression. *GAPDH* was used as a control for quantitative analysis of *Idh2* and *Cdk2* expression. Data are expressed as means ± SD (n = 3).

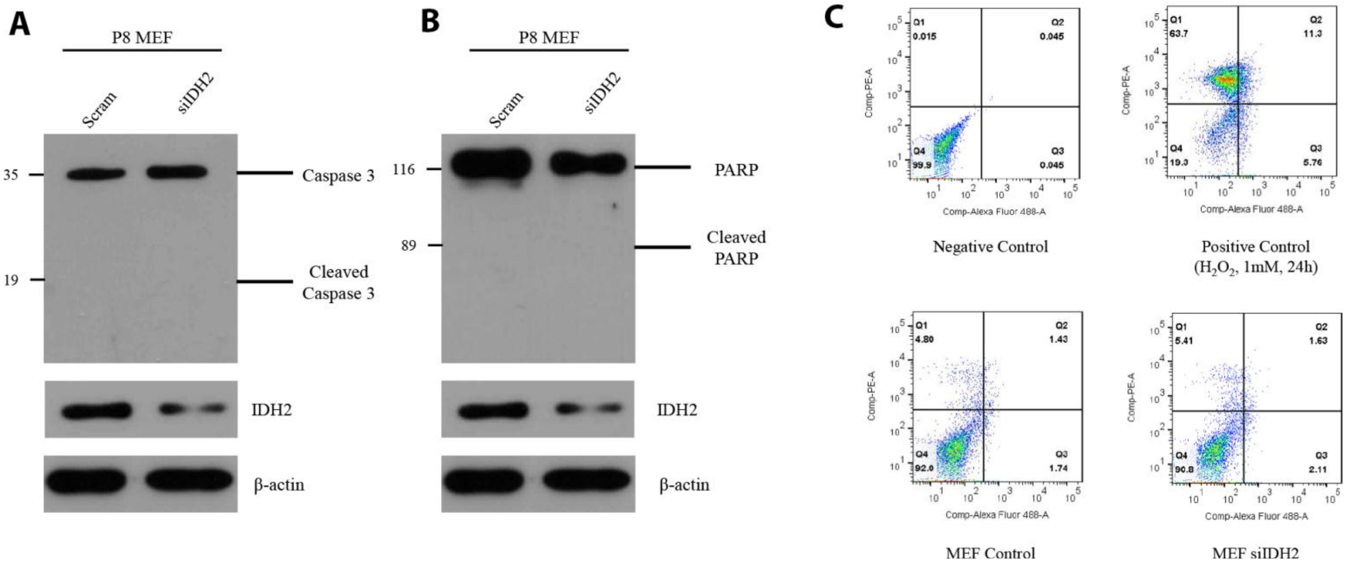

Supplementary Figure 4. *Idh2* silencing did not affect the induction of the apoptotic signaling pathway in mouse embryonic fibroblasts (MEFs). Western blot analysis of apoptotic marker protein caspase 3 (**A**) and PARP (**B**) in control and *Idh2*-knockdown MEFs. Passage 8 (P8) MEFs were used for siRNA transfection. Membranes were blocked in bovine serum albumin (BSA), and first antibodies were diluted 1:1000 in BSA. β-actin was used as a control for quantitative analysis of Caspase 3 and PARP. (**C**) Flow cytometry of the apoptotic effect of *Idh2*-knockdown in MEFs. After treatment with siRNA for 24 h, cells were harvested and stained with Annexin V, followed by FACS analysis (PE-A : PI staining / AlexaFluor 488-A : Annexin V staining).
